# Delayed Diagnosis of an Aspirated Dental Crown Presenting as Chronic Bronchitis and Pneumonia in an Adult Male: A Case Report

**DOI:** 10.7759/cureus.91114

**Published:** 2025-08-27

**Authors:** Nabil Tiresse, Soufiane Elfathi, Adil Zegmout, Hanane Elouazzani, Ismail Rhorfi

**Affiliations:** 1 Department of Pneumology, Mohammed V Military Training Hospital, Rabat, MAR

**Keywords:** chronic bronchitis, dental crown, foreign body aspiration, pneumonia, pulmonary parenchymal destruction

## Abstract

Foreign body aspiration is uncommon in adults compared to children. The aspiration of dental materials is a relatively rare event, whether it occurs during, or independently of, dental procedures. Its true incidence remains difficult to assess due to variations across reported studies. Such aspirations can lead to both immediate and delayed complications, including pneumonia, which may result in local or systemic consequences.

We report a rare case of an unnoticed aspiration of a dental crown, discovered several months after inhalation. A 44-year-old male with a history of chronic tobacco use presented with chronic cough, recurrent episodes of bronchitis, and general health deterioration. Chest X-ray (frontal and lateral views) and thoracic computed tomography (CT) guided the diagnosis, which was subsequently confirmed via flexible bronchoscopy. Interestingly, the dental crown was spontaneously expectorated by the patient a few hours after the bronchoscopy.

Follow-up imaging revealed persistent, irreversible parenchymal destruction, despite clinical improvement and resolution of recurrent bronchitis. From a theoretical standpoint, and according to the literature, rigid bronchoscopy remains the gold standard for the removal of aspirated dental materials. This case underscores the importance of considering foreign body aspiration in the differential diagnosis of recurrent respiratory infections and chronic bronchitis. It also highlights the value of standard chest imaging in the initial assessment, as well as the utility of flexible bronchoscopy outside of the acute setting.

## Introduction

Foreign body aspiration is a common reason for pediatric emergency consultations. In adults, however, such cases are relatively rare [1]. The aspiration of dental materials, whether occurring during or outside of dental procedures, is even less frequently reported [2]. We present the case of a chronic smoker who had been experiencing recurrent episodes of bronchitis over several months. The underlying cause was eventually identified as the unnoticed aspiration of a dental crown, which led to localized parenchymal lung destruction. To our knowledge, this represents one of the few documented cases of pneumonia with pulmonary parenchymal damage caused by the aspiration of a dental prosthesis. This case highlights a rare pathology, whose diagnosis was delayed due to misinterpretation of initial symptoms as post-tobacco bronchitis.

## Case presentation

We report the case of a 44-year-old male with a 20-pack-year history of chronic tobacco use. For more than six months, he had experienced a persistent cough, accompanied by mucous and, at times, purulent sputum. These symptoms were unresponsive to empirical antibiotic therapy and symptomatic treatment. Six months earlier, the patient underwent dental treatment involving the placement of a dental crown, which was performed correctly, without any immediate complications. The patient had no history of surgery, psychiatric disorders, or other significant medical conditions, and, specifically, no diagnosis of chronic obstructive pulmonary disease (COPD). There was no personal or family history of neoplastic or genetically linked pulmonary disease. He had mistakenly attributed his symptoms to post-smoking chronic bronchitis. The patient also reported unquantified weight loss over the same period, without any associated extrapulmonary symptoms.

**Figure 1:**
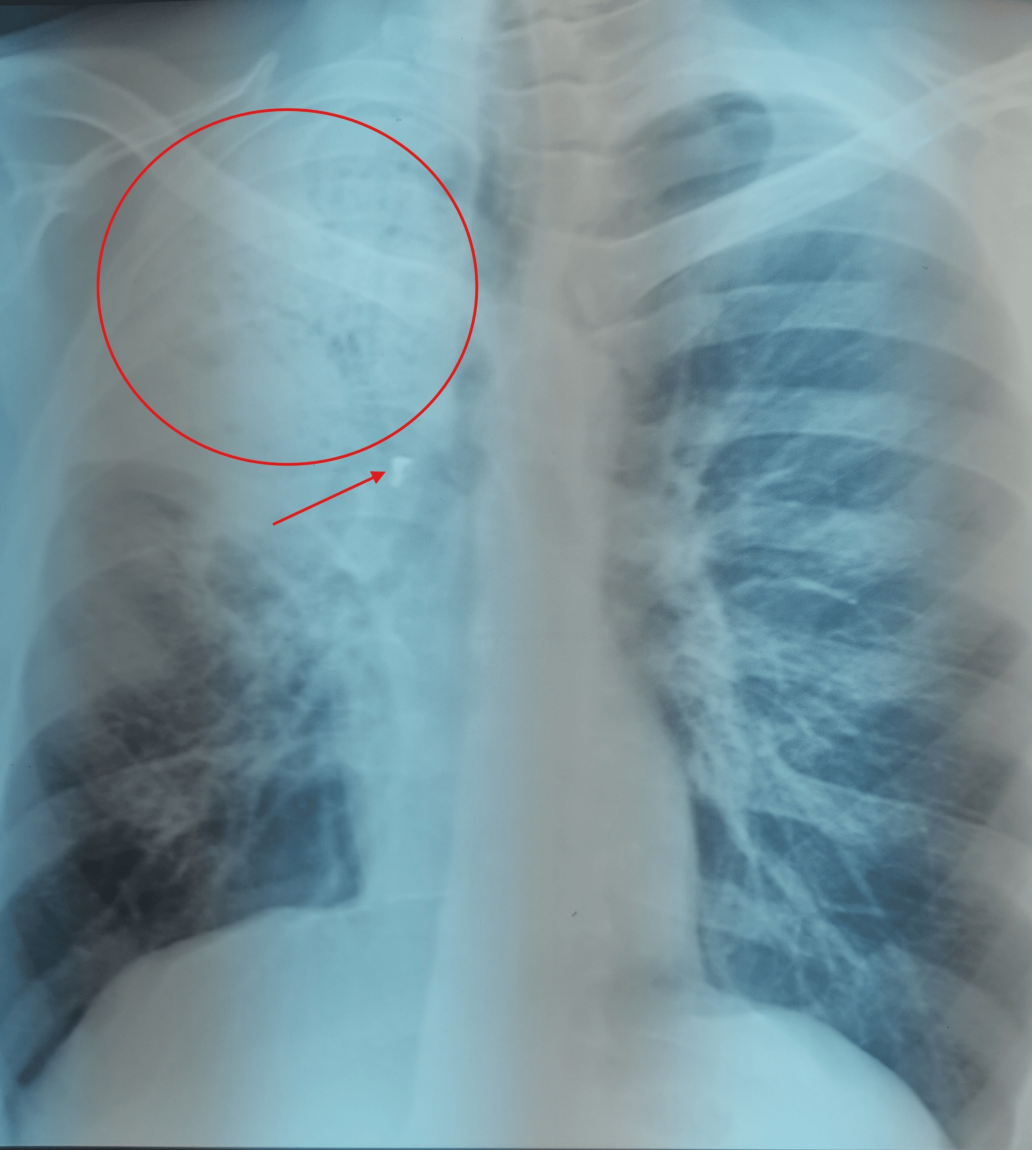
Frontal chest X-ray showing acute lobar pneumonia (red circle) with a calcified opacity projected over the right upper lobe bronchial orifice (red arrow).

On physical examination, signs of pulmonary consolidation were observed in the upper half of the right hemithorax. No wheezing was noted during either phase of respiration. Intraoral examination revealed poor dental hygiene; the teeth had obvious caries. A frontal chest X-ray showed an acute lobar pneumonia involving the upper third of the right lung, along with a calcified opacity at the entry of the right upper lobar bronchus (Figure 1).

**Figure 2:**
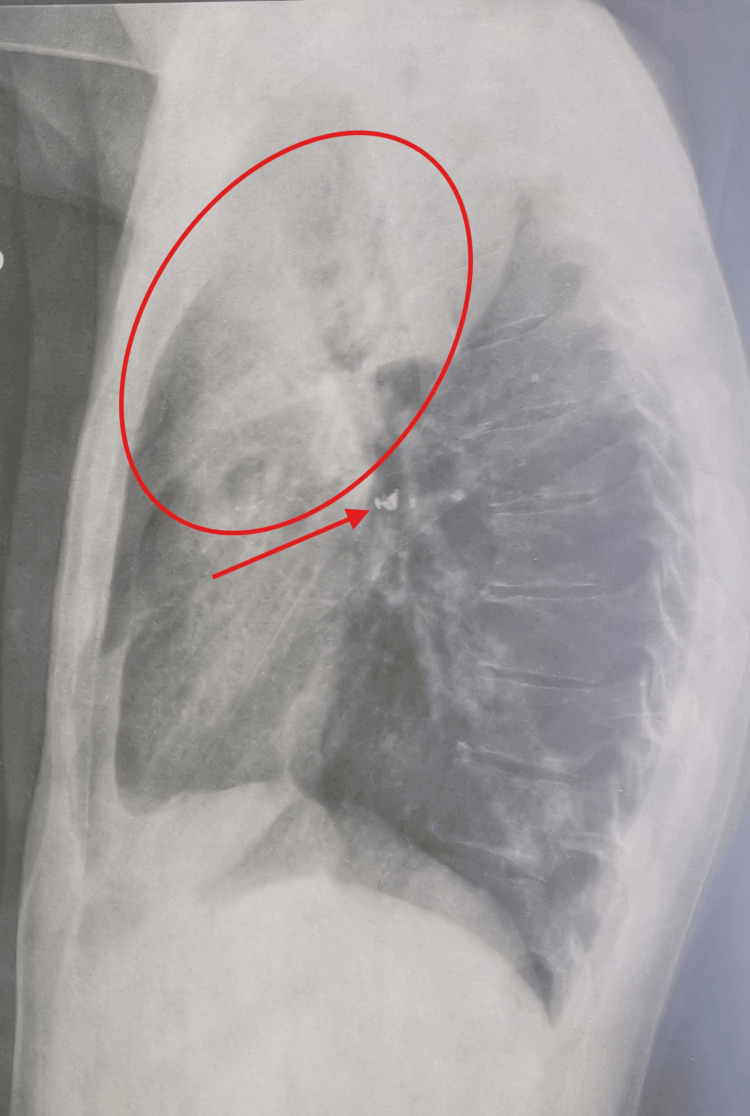
Lateral chest X-ray confirming the presence of a calcified opacity projected over the right upper lobe bronchus (red arrow), associated with right upper lobe consolidation (red circle).

A lateral X-ray view confirmed the right upper lobe involvement and the presence of a similar calcified opacity within the corresponding bronchus (Figure 2).

**Figure 3:**
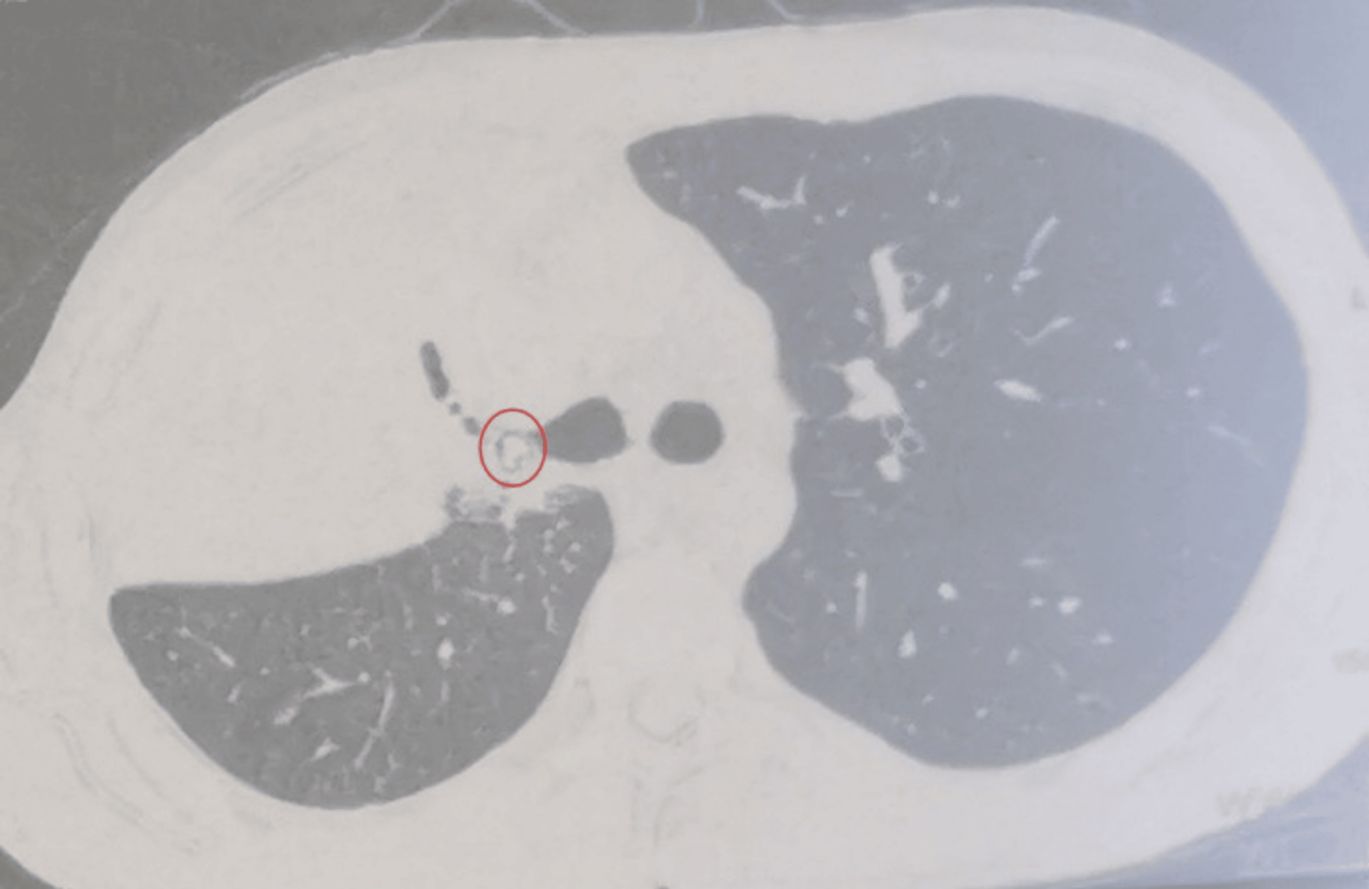
Axial CT scan showing a foreign body in the right upper lobe bronchus (red circle), associated with alveolar consolidation of the same lobe. CT, computed tomography

Chest computed tomography (CT) scan revealed a right upper lobar alveolar consolidation containing an air bronchogram distal to a calcified nodule located within the right upper lobar bronchus (Figure 3).

**Figure 4:**
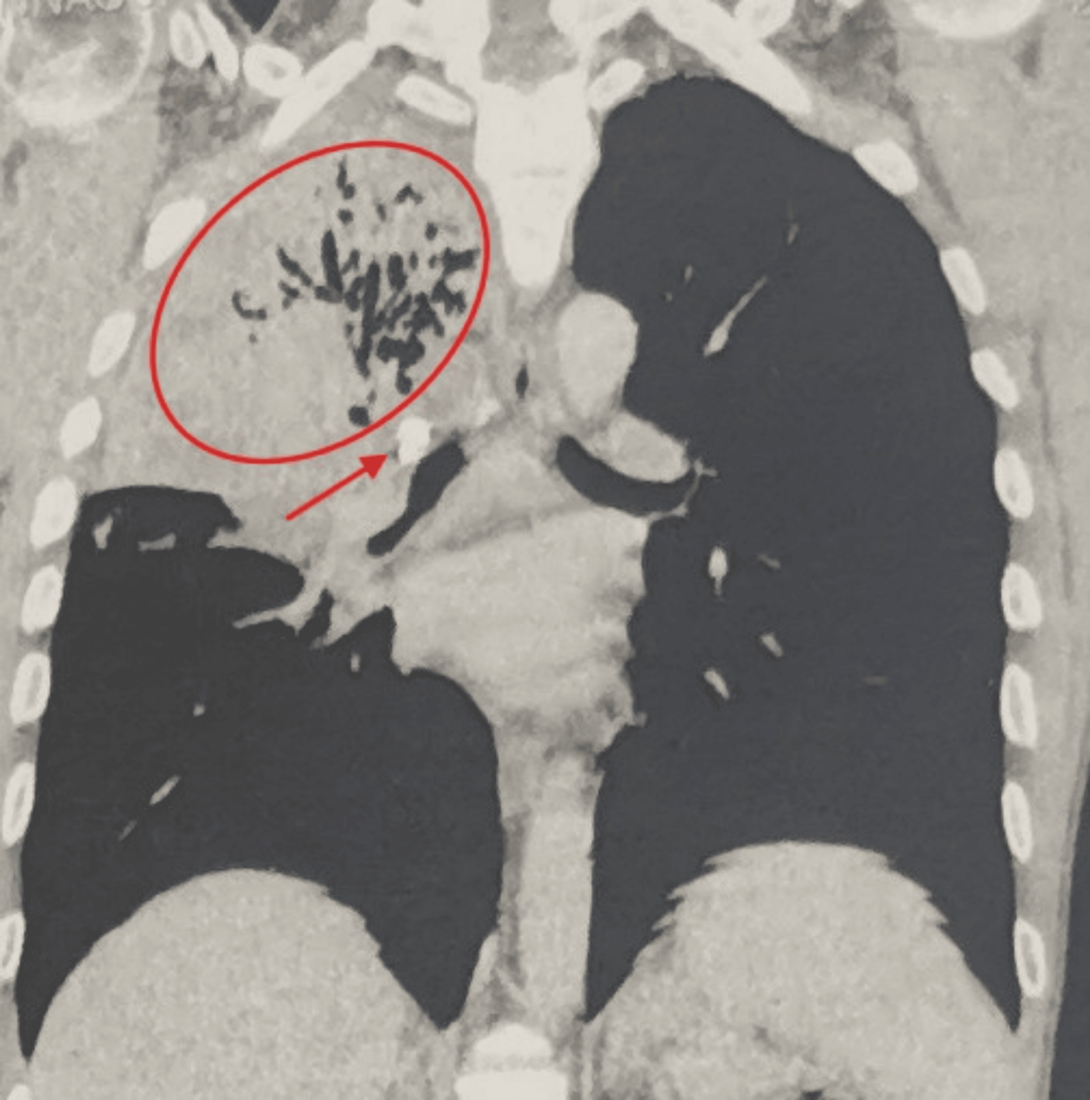
Coronal CT scan showing right upper lobe alveolar consolidation (red circle) with a calcified opacity in the right upper lobar bronchus (red arrow). CT, computed tomography

Coronal slices confirmed the presence of lobar pneumonia with parenchymal destruction distal to a rounded lesion that nearly completely obstructed the entrance to the bronchus (Figure 4). Bacteriological analysis of sputum samples did not isolate any specific pathogens.

Given the patient’s history of chronic smoking, general health deterioration, and thoracic imaging findings, infectious pneumonia - particularly tuberculosis - and neoplastic processes were included in the differential diagnosis.

**Figure 5:**
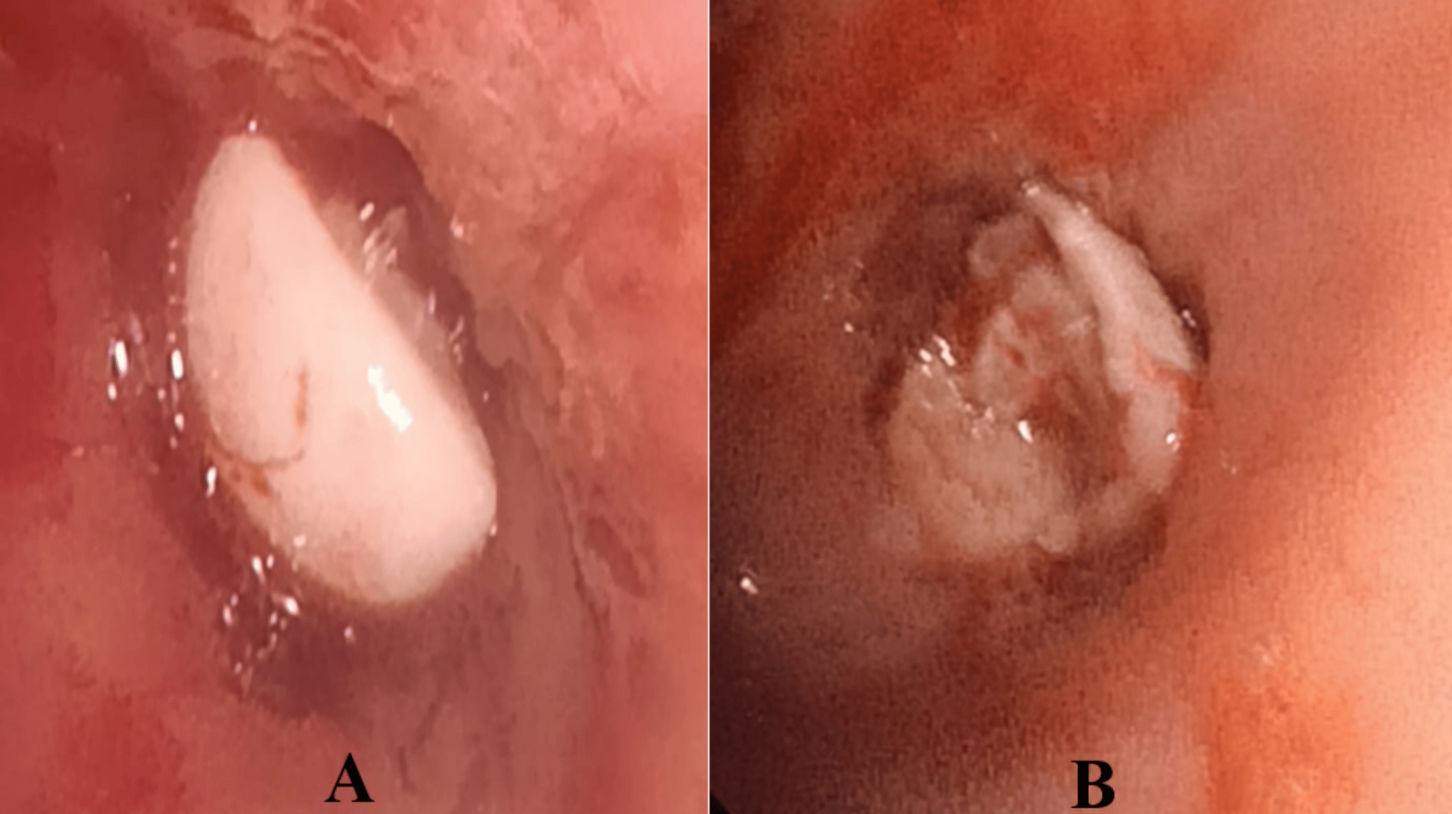
Bronchoscopic examination showing a foreign body at the entrance of the right upper lobar bronchus, with a smooth surface on one side (A) and an irregular surface on the other (B).

**Figure 6:**
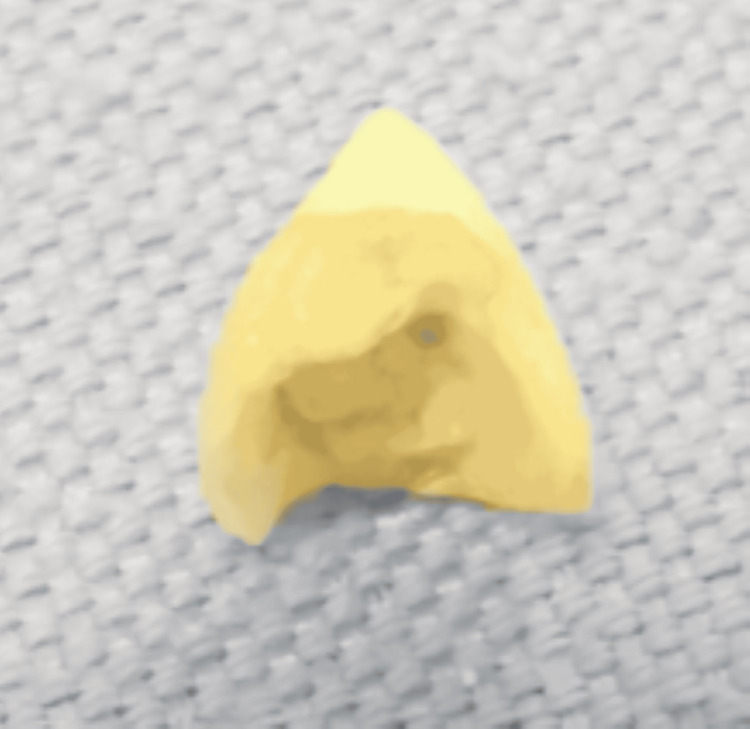
The foreign body expectorated by the patient and identified as a dental crown.

A diagnostic flexible bronchoscopy was performed, revealing a foreign body at the entrance of the right upper lobar bronchus, with a smooth surface on one side (Figure 5A) and an irregular surface on the other (Figure 5B). Attempts to extract the lesion during bronchoscopy were unsuccessful. Three days later, during a coughing episode, the patient spontaneously expectorated the foreign body (Figure 6), which was subsequently identified as a dental crown.

**Figure 7:**
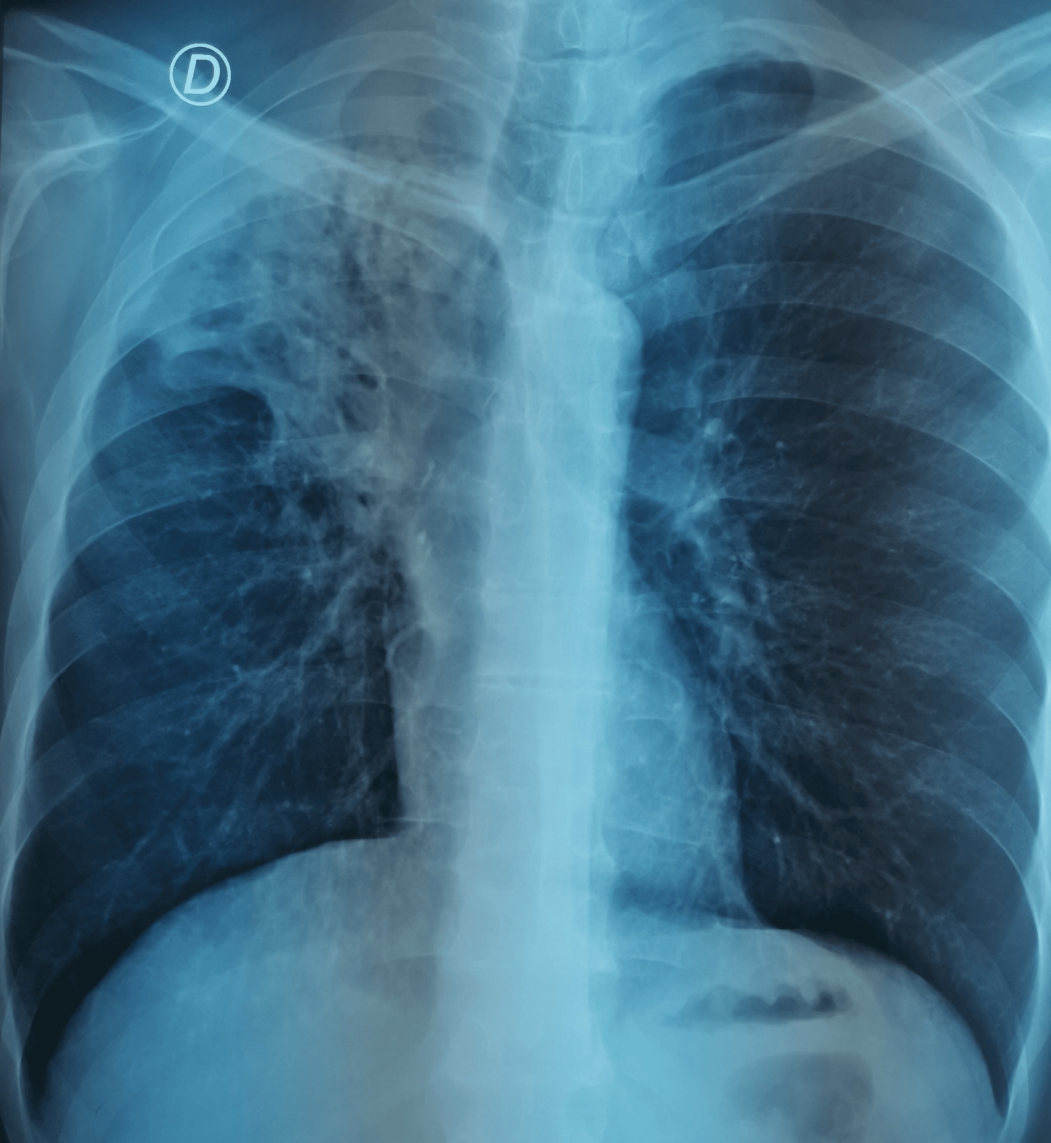
Follow-up frontal chest X-ray showing no residual endobronchial foreign body; persistent right upper lobe alveolar syndrome.

*Haemophilus influenzae* was isolated from the bronchial aspirate, and the patient was treated with amoxicillin-clavulanic acid for seven days in accordance with the antibiogram results. A follow-up chest X-ray performed two weeks after bronchoscopy showed persistent right upper lobar alveolar consolidation with slight regression (Figure 7). No residual foreign body was observed at the right upper lobar orifice, the intermediate bronchus, or the right lower lobar bronchus.

The patient reported significant clinical improvement, including resolution of cough and purulent sputum. He also experienced weight gain, coinciding with the initiation of smoking cessation at the beginning of his care. Annual chest imaging is recommended to monitor for potential complications, particularly aspergilloma formation or neoplastic transformation. Influenza and pneumococcal vaccinations were prescribed to prevent severe bacterial or viral bronchitis in the future. Surgical intervention via lobectomy remains a potential therapeutic option in the event of recurrent infections or complications such as hemoptysis or fungal formation.

## Discussion

Foreign body aspiration is frequently observed in pediatric emergency settings, and delayed intervention may lead to serious health complications, including increased morbidity and mortality [1]. In adults, foreign body aspiration is rarely reported, and when it does occur, it most often involves food-related respiratory distress episodes [1]. In a study conducted in France over 11 years involving a sample of 24,651 cases, the incidence of dental material aspiration was estimated at 44 cases [2]. Another study conducted in Tokyo reported four cases of aspiration among 40 incidents of ingestion or aspiration [3]. Advanced age, alcoholism, mealtimes, and poor dentition have been identified in the literature as major risk factors for foreign body aspiration [1,4]. In odontological practice, additional risk factors for aspiration may include the supine position, small instrument size, limited visual access, and handling slippery prosthetic components [5,6]. Dental crowns, commonly used in fixed prosthodontics, are frequently involved [7,8]. 

Dyspnea, paroxysmal coughing, and cyanosis are the main symptoms of foreign body aspiration [1,9]. These symptoms can progress to respiratory distress [10,11]. However, foreign body aspiration may be entirely asymptomatic in two-thirds of cases [8], as was observed in our patient. This often leads to delayed diagnosis, particularly when clinical manifestations are nonspecific [4]. Such diagnostic delays significantly increase the risk of complications, including bronchial obstruction and parenchymal destruction [6,10].

The time to diagnosis can vary widely and is primarily influenced by the initial symptomatology, as well as the attentiveness of the dental surgeon and their assistant [5]. In cases of silent aspiration, this delay may extend over several years [8]. Exploring other differential diagnoses can sometimes delay the final diagnosis, as clearly illustrated by our case. The presence of chronic cough in our patient, who had no known history of COPD, led us to revise the diagnosis.

The most direct radiologic sign of foreign body aspiration is the visualization of an endobronchial radiopaque object, as seen on the standard chest X-ray of our patient (Figures 1-2) and on the CT scan slices (Figures 3-4), which applies to the majority of high-risk items encountered in dental practice [5]. Atelectasis, pneumonia, lung abscess, bronchiectasis, or empyema are other indirect radiologic findings [11]. In our patient, the presence of right upper lobe pneumonia distal to a calcified opacity at the entrance of the corresponding bronchus strongly suggested a foreign body aspiration complicated by secondary infection (Figures 1-4).

Body position may influence the location of intrabronchial dental material [6]. It is most frequently found in the right lower lobe bronchus or the right main bronchus [8,11]. In some patients, no lesions are visible on standard chest radiographs [12]. Although chest CT is often not required for initial diagnosis, it is typically performed when complications are suspected or in cases of diagnostic doubt [6,8].

The combination of clinical and radiological findings is often sufficient to guide the diagnosis [13]. After chest imaging, bronchoscopy is necessary both to confirm the diagnosis and to remove the foreign body [6]. Louhaichi et al. conducted a study involving 105 children, which demonstrated the usefulness of flexible bronchoscopy in foreign body extraction [14]. As commonly observed in our patient, flexible bronchoscopy allowed visualization of the foreign body, whose mobilization facilitated its extraction following coughing efforts after bronchoscopy (Figure 5). Other reports have highlighted the value of virtual bronchoscopy, particularly in complex cases or when the foreign body is radiolucent on chest radiography [11,12]. Virtual bronchoscopy has not yet been implemented in our department.

Therapeutically, prompt and appropriate management is essential to prevent the immediate and delayed complications of foreign body aspiration, particularly when dental materials are involved. During dental care, healthcare professionals should be able to recognize the acute clinical signs of foreign body aspiration [5]. The initial approach should focus on ensuring adequate pulmonary oxygenation. In cases of life-threatening respiratory distress, emergency interventions such as the Heimlich maneuver, endotracheal intubation, or tracheotomy may be required [8]. This highlights the need to have reanimation equipment available during dental care for patients with aspiration risk factors.

In cases of foreign body aspiration, rigid bronchoscopy should be performed without delay, in collaboration with pulmonologists, radiologists, and anesthesiologists [13]. When rigid bronchoscopy fails, surgical intervention becomes necessary. Depending on the extent of damage and the location of the foreign body, bronchotomy, thoracotomy, and lobectomy are the most commonly used techniques [8].

In the present case, the patient spontaneously expelled the dental crown three days after flexible bronchoscopy during a coughing episode (Figure 6). However, persistent parenchymal damage was noted, posing a potential source of future complications such as recurrent infection, hemoptysis, aspergilloma formation, or even malignant transformation - especially in the context of chronic tobacco use (Figure 7). Surgical management via lobectomy is currently being considered to minimize these risks.

Preventing foreign body aspiration during dental care remains a priority by reducing the incidence of such events, which can sometimes lead to severe complications. Araujo et al. proposed the use of rubber dams during dental procedures [5]. Special attention should be given during complex dental procedures, such as dental implant prosthesis.

One of the strengths of this case lies in the timely diagnosis, despite the absence of an acute clinical context suggestive of dental material aspiration. However, this report also highlights limitations. Notably, the absence of a regional or national registry for such events makes it difficult to accurately assess their epidemiological frequency and to implement large-scale preventive strategies aimed at minimizing the risk of similar incidents. Additionally, the omission of rigid bronchoscopy from the management protocol could have had detrimental consequences if the dental crown had not been spontaneously expelled.

## Conclusions

Foreign body aspiration in adults is an uncommon but potentially serious clinical event, particularly when involving dental materials. This case underscores the diagnostic challenges associated with asymptomatic or non-specific presentations, which may mimic chronic bronchitis or recurrent infections - especially in smokers. Clinical signs, imaging, and bronchoscopy are sufficient to establish the diagnosis. Rigid bronchoscopy remains the gold standard for management, but flexible bronchoscopy may offer diagnostic and therapeutic benefits in selected cases. Attention should be focused on the complications arising from delayed management. Finally, dental surgeons must promptly identify and manage acute foreign body aspiration and educate patients to seek care if unusual respiratory symptoms arise. Multidisciplinary management is important for improving patient outcomes.
